# Supplementary figures and images for: Establishment of a Pseudovirus Platform for Neuraminidase Inhibiting Antibody Analysis
Source: Int J Mol Sci. 2023 Jan 25;24(3):2376. doi: 10.3390/ijms24032376 (PMC9916614; doi:10.3390/ijms24032376)

A

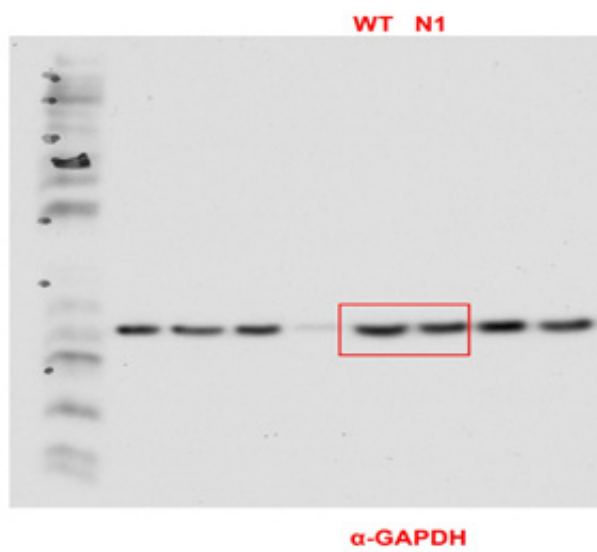

B

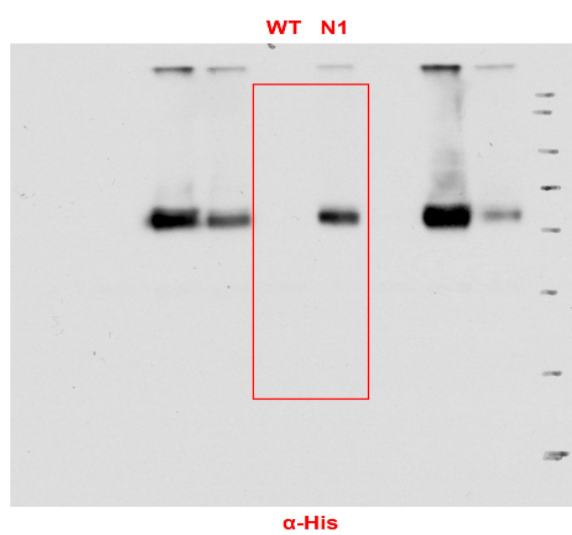

Supplementary S1. The original images of western blots.

Supplement: Supplementary file 1 [file ijms-24-02376-s001.zip › ijms-2145160-supplementary.pdf]
